# Perceptual shape sensitivity to upright and inverted faces is reflected in neuronal adaptation

**DOI:** 10.1016/j.neuroimage.2009.12.077

**Published:** 2010-04-01

**Authors:** Sharon Gilaie-Dotan, Hagar Gelbard-Sagiv, Rafael Malach

**Affiliations:** a Department of Neurobiology, Weizmann Institute of Science, Rehovot 76100, Israel; b Wellcome Centre for Neuroimaging, University College London, London, UK; c Institute of Cognitive Neuroscience, University College London, London, UK

**Keywords:** Face perception, fMRI, Human cortex, Object recognition, Ventral stream

## Abstract

Using an fMR-adaptation paradigm for different face morphing levels we have recently demonstrated a narrow neuronal tuning to faces even at the sub-exemplar level which was tightly related to perceptual discrimination (Gilaie-Dotan and Malach, 2007). However, it is unclear whether this relationship is unique to faces or is a general property of object representations including unfamiliar objects, and whether the adaptation tuning is due to physical changes in the stimulus or to changes in perceptual discrimination. Here we compared the same face-morph paradigm for upright and inverted faces, thus modulating familiarity and perceptual discrimination effects while equating all low-level features. We found, as expected, a perceptual “inversion effect”, i.e. a significant reduction in inverted face discrimination. Importantly, the fMR-adaptation tuning in the fusiform face area (FFA) changed in accordance with the different perceptual sensitivity both for upright and inverted faces. Additional object selective regions displayed differential tuning widths to the two categories. Our results are compatible with a model by which the ability of human observers to discriminate objects depends on the shape tuning properties of individual neurons.

## Introduction

Human perception manifests a remarkable capacity for discriminating subtle shape changes in visual objects while maintaining a robust invariance for other optical parameters such as position, size and contrast. A large body of research has by now demonstrated that these invariances are accomplished gradually along the visual hierarchy leading from primary, retinotopic visual areas—which are sensitive to low-level parameters such as position (Grill-Spector et al., 1998; Sereno et al., 1995), size and contrast (Avidan et al., 2002a), to high order visual areas which show a reduced sensitivity to low-level, local image features, yet exhibit enhanced sensitivity to more holistic aspects of the stimulus such as shape changes (Grill-Spector et al., 2001).

In particular, high order object-related regions often display object category selectivity—with prominent examples of face-related (Allison et al., 1994; Ishai et al., 1999; Kanwisher et al., 1997; Levy et al., 2001; Puce et al., 1995), place-related (Aguirre et al., 1998; Epstein and Kanwisher, 1998; Ishai et al., 1999; Levy et al., 2001, 2004a), and more general object-related categories (Grill-Spector, 2003; Grill-Spector et al., 2001; Hasson et al., 2003; Malach et al., 1995; Tootell et al., 1996).

While a large body of experimental data has accumulated regarding the various aspects of human object representations, an important, still unresolved question concerns the role of *individual neurons* in determining the remarkable behavioral shape sensitivity of human observers. In particular, it is still not clear whether human shape sensitivity is already present in the shape tuning of individual neurons or whether it is a product of a joint population activity while the individual neuronal selectivity is far coarser.

**Fig. 1:**
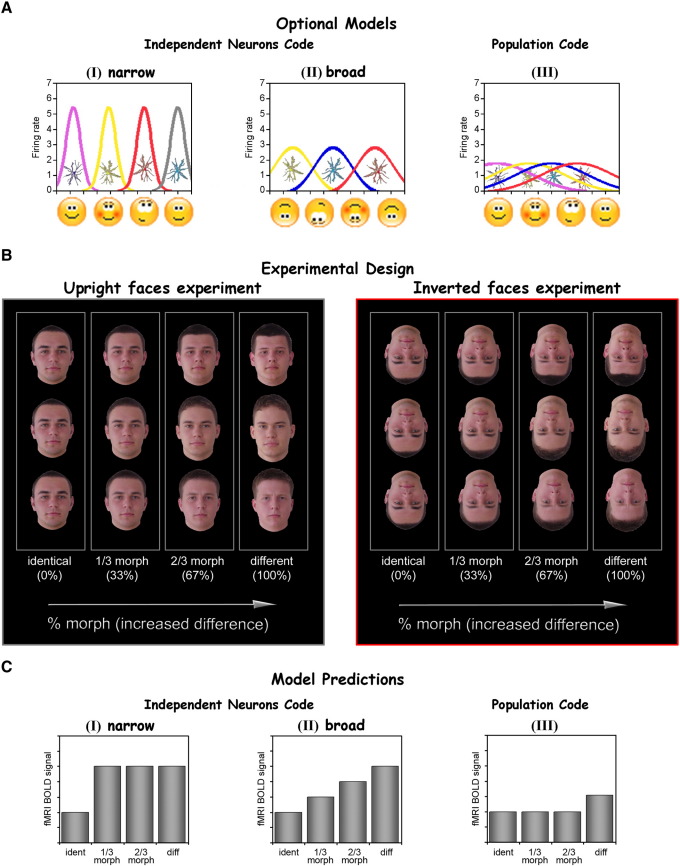
Shape tuning of individual neurons: optional models, experimental design, and model predictions. (A) Two different models for the role of individual neurons in determining the behavioral shape sensitivity of human observers. In the “independent-neurons code” model the behavioral shape sensitivity is already present at the level of individual neurons. Neurons can be either narrowly tuned (I) or broadly tuned (II), corresponding to the behavioral shape discrimination ability of the observer. Alternatively, in a “population code” (III) model the behavioral sensitivity of the observer is a product of the relationship between neurons whose individual selectivity is far coarser. The colored curves represent the shape tuning curve of individual neurons (firing rate on the *Y* axis, distance between objects on the *X* axis). (B) Experimental design of the morph experiment for upright faces (left) and inverted faces (right). Faces were morphed into several different face exemplars. The morph sequences are represented along the *X* axis. Faces at four stages along the morph sequence (identical, 1/3 morph, 2/3 morph and different) were used to build the four different conditions for the block design experiments. (C) Model predictions according to the experimental design laid out in (B). In a narrow independent-neurons model (I), a slight shift in face shape (1/3 morph, see (B)) would be sufficient to move the activation to a different set of neurons leading to a full release from adaptation. In the broad independent-neurons model (II) recovery from adaptation requires a larger shape change. Finally, in the population model (III) the neuronal tuning can be extremely broad requiring a change in object category (category tuning) to achieve recovery from adaptation.

To illustrate this issue more concretely, consider two extreme alternatives that can be envisioned in the relationship between individual neurons' shape tuning and the psychophysical recognition performance. In the first model, akin to the extensively discussed “population vector” concept, the shape tuning curves of the neurons are broad and overlapping. The tuning curve of each neuron in such a model is far coarser than the actual discrimination performance of the observer. Narrow behavioral tuning can then be computed by using the *relationship* between individual neurons, e.g. the ratio of firing rates, as the code of the represented object. A canonic example of such vector representation is that of color coding where activity in three broadly tuned and overlapping color sensitive receptors generates a representation of numerous narrowly tuned hues. Similar population coding schemes have been proposed for direction of motor movement generation in the primate cortex (Georgopoulos et al., 1986) and for motion detection and discrimination in MT+ (Jazayeri and Movshon, 2006). More recently the notion of broadly tuned neuronal representations was extended to faces, both in fMRI (Jiang et al., 2006), and in single cell recordings of monkeys (Freiwald et al., 2009). A schematic illustration of this model is presented in Fig. 1A (III) and we will refer to it as the “population tuning” coding model. At the other extreme, one could envision a scheme in which the shape tuning sensitivity of the observer is derived from the tuning of *individual* shape-selective neurons. In such shape tuning model the limiting factor on the discrimination performance is the independent tuning curves of individual neurons and no additional “sharpening” is achieved by the group relationships among these different neurons. A paradigmatic example of this kind of model with “narrow tuning” is the cochlear representation of sound frequencies. Intriguingly, such hyper-fine tuning of single cortical neurons that matches human sound discrimination has recently been discovered also in single neuron recordings from human auditory cortex (Bitterman et al., 2008). This model is illustrated in Figs. 1A (I) and (II) and we will refer to it as the “independent neurons tuning” coding model.

It is important to emphasize that the independent neurons tuning model *does not* imply a “grandmother” type scheme—in which only few neurons are activated by each object image. To the contrary, even in an independent neurons tuning scheme a large neuronal population might be activated by each object image. The difference between the two models is rather in the *source* of the behavioral shape sensitivity. While in the population model this is an emergent property of the inter-relationships within the population, in the independent neurons model the behavioral shape sensitivity is a direct reflection of the tuning properties of the individual neurons. We elaborate further on these points in the discussion.

An important prediction that could easily distinguish between these two alternative models is as follows: in the independent tuning model, stimuli for which human observers show fine or coarse discriminations should have neuronal representations manifesting narrow or coarse tuning respectively. These alternatives are illustrated as “narrow” and “broad” tuning in Figs. 1A (I) and (II). In contrast, no such direct relationship is expected from the population coding model, since here it is the *relationship* between the neurons rather than their independent tuning selectivity that matters. Rather, in a population model the reduced selectivity is typically due to a reduction in the number of channels—e.g. the loss of a color pigment associated with “color blindness”.

When employing the method of BOLD fMRI, it is impossible to directly examine the shape selectivity at the level of individual neurons since the BOLD signal represents the averaged activity of a large number of neurons. A possible means for targeting the neuronal responses may be offered through adaptation (also termed repetition suppression) effects (Grill-Spector and Malach, 2001). By repeatedly presenting a single object image, one presumably could target the neurons that are selectively activated by this object image and suppress their activation (i.e. the adaptation baseline). If we then parametrically change the shape of the object (e.g. through morphing), we may reveal, at least qualitatively, the sensitivity (tuning) of the neuronal population specifically engaged in representing that aspect of the object image (evident by release from adaptation).

In a previous research, using upright morphed faces (see Fig. 1B left panel) and an fMRI-adaptation paradigm, we found that cortical face representations appear to follow the independent neurons coding (Fig. 1C (I)) showing a strikingly narrow, sub-exemplar tuning as revealed by neuronal adaptation (Gilaie-Dotan and Malach, 2007). This narrow neuronal tuning matched quite well the behavioral discrimination performance of subjects.

However, given the special status of upright faces (Farah, 1996; Farah et al., 1998; McKone et al., 2007), it could be the case that faces engage specialized representations which do not generalize to other shape categories.

In the present study we thus used fMR-adaptation effects to compare shape tuning for upright faces with that for *inverted faces* (see Fig. 1B) across different regions in object-selective cortex. The advantage of using upright and inverted faces is three-fold: (i) manipulating performance: it is well established that the human discrimination performance is disproportionately impaired for inverted compared to upright faces. This effect, termed the face inversion effect (FIE), has been extensively studied and documented (e.g. (Farah et al., 1995; Goffaux and Rossion, 2007; Murray et al., 2000; Rhodes et al., 1993; Rossion and Gauthier, 2002; Valentine, 1988; Yin, 1969; Yovel and Kanwisher, 2005)); (ii) manipulating familiarity: faces are one of the categories which humans are known to be highly familiar with whereas inverted faces are an opposite extreme with humans having no or very limited experience with; (iii) controlling for physical differences: In contrast to this robust behavioral difference between upright and inverted faces, the low level feature composition of upright and inverted faces is identical.

A prediction of the independent neurons code, is that the behavioral difference in shape sensitivity that exists between upright and inverted faces (the FIE) should lead to different profiles of adaptation for upright and for inverted faces. These predictions are shown in Fig. 1C (left panels I and II). In the population code scheme on the other hand, one would expect a broad adaptation profile regardless of face orientation (Fig. 1C (III)), with perhaps a higher level of activation for the upright faces.

Our results replicate our previous findings of narrow tuning to upright faces (Gilaie-Dotan and Malach, 2007). However, for inverted faces, we find a significantly broader adaptation profile in the FFA. Importantly, this broadening nicely correlates with the reduced perceptual ability to detect shape differences in these images, thus supporting a model of independent-neuronal code in the human FFA.

## Materials and methods

### Subjects

12 healthy subjects (6 women, ages 22–31, average age 28) participated each in the three fMRI experiments (upright morph, inverted morph, and visual localizer) and in two behavioral experiments (upright morph and inverted morph) that took place outside the scanner following the fMRI scan (on the same day). All subjects underwent a short training session of 2 minutes prior to the scan on a different set of stimuli. All subjects had normal or corrected-to-normal vision. The experiments were undertaken with the understanding and written informed consent of each subject to participate in the fMRI experiments. The Tel Aviv Sourasky Medical Center approved the experimental protocol.

### Magnetic resonance imaging setup and acquisition

Scanning was done on a Siemens MAGNETOM Trio A Tim System 3T scanner, equipped with a standard Siemens head coil, at the Helen and Norman Asher Center for Human Brain Imaging in the Weizmann Institute of Science. Blood oxygenation level-dependent (BOLD) contrast was obtained with gradient-echo echo-planar imaging (EPI) sequence. The time repetition (TR) used was 3000 ms, echo time (TE) = 30 ms, flip angle = 90°, field of view = 240 × 240 mm^2^, matrix size = 80 × 80, the scanned volume consisted of 46 nearly axial slices of 3-mm thickness (no gap) with an in plane resolution of 3 × 3 mm^2^, covering the entire cortex. A whole-brain T1 magnetization prepared rapid acquisition gradient echo (MPRAGE) sequence was acquired on each subject to allow accurate cortical segmentation, reconstruction, and volume-based statistical analysis. Here the TR was 2300 ms, TE = 2.98 ms, flip angle = 9°, field of view = 256 × 256 mm^2^ and the scanned volume consisted of 160 slices of 1 mm thickness with 0.5 mm gap, and in plane resolution of 1 × 1 mm^2^. When this detailed anatomical scan was not performed next to the functional experiment, a shorter and less detailed T1 MPRAGE (96 slices of 2 mm thickness and 1 mm gap, TE = 2.67 ms and the rest of parameters identical) was performed and was automatically (Brain Voyager QX) coregistered to the more detailed anatomical scan. Button presses were recorded during the fMRI experiments via a response box (Current Designs MR safe fiber optic response pad, bimanual 8 model) from which we analyzed the behavior performance. Due to technical reasons in one subject we failed to collect behavior data during the inverted faces fMRI experiment.

### Stimuli

Stimuli were generated on a PC, projected via an LCD projector (Epson PowerLite 74c) onto a screen positioned at the back end of the MRI tunnel, and viewed through a tilted mirror, positioned over the subject's forehead. Stimuli were based on 78 original different color photographs of male faces taken from 2 databases (mainly CVL Face Database (http://www.lrv.fri.uni-lj.si/facedb.html) and also AR Face Database (Martinez and Benavente, 1998)). Frontal images were chosen of mostly Caucasian, with neutral expression, mouth closed, no facial hair, and no glasses. The original images were then processed using Adobe Photoshop 6 in the following manner: rotated to upright, such that the line connecting the eyes was horizontal; aligned to each other by rescaling to a common face size and location and then aligned by the middle vertical line crossing the nose of each face and by the horizontal line that passes below the eyes; the background was set to black, and the neck was cropped naturally (as if wearing high-neck black shirt); hairstyle was set above the ears and images were cropped to 600 × 600 pixels (10° × 10°).

The morphing of the original images was done using MorphMan 4.0 (STOIK Imaging, Moscow, Russia). The morphing was done in sets of 13 different original images, where one face (source face) was morphed to the other 12 different faces (target faces) (Gilaie-Dotan and Malach, 2007). The main alignment features for the morph included hairline, lips, nose, eyes, eyebrows, and the external contour.

A black image (matching the black background of the face stimuli) was used during the fixation periods. A red fixation dot of 4 × 4 pixels (0.07° × 0.07°) appeared at the center of the screen throughout the experiment.

### Block design upright and inverted morph fMRI experiments

The experiment lasted 480 s and included 4 conditions: identical, 1/3 morph, 2/3 morph, and different. Each condition was repeated 6 times in a controlled and counterbalanced block design paradigm. Each block lasted 12 s, with interleaving 6-s fixations between blocks. The first and last fixations lasted 21 and 15 s, respectively. A block consisted of 12 different stimuli; each stimulus was presented for 1000 ms.

Consecutive images were slightly shifted in an equally balanced manner (across conditions, across Euclidean distance) in all conditions to avoid motion cue confounds and to eliminate tactics of retinal differences. All 11 translations within a block were equal in size, following a translation path along 12 symmetrical points (with regard to the *x* axis and *y* axis) in a 2D square (maximum size 0.57° × 0.57°). Average translation between 2 consecutive images over all translations in the experiment was 0.24° (minimum 0.15°, maximum 0.32°). The average Euclidean distance of the shifted images was as follows—identical: 23.60, 1/3 morph: 22.80, 2/3 morph: 21.54, and different: 26.23. Average Euclidean distance in a block across the whole experiment was 23.49 (see details below). Subjects' task was to fixate and respond via a response box whether a face image was the same face image (should report “same”) or a different face image (should report “different”) than the previous one.

To allow for a 1-back recognition task, in the identical condition, we introduced occasionally a matching 1/3 morph image (of the 6 identical blocks, 2 blocks contained no 1/3 morph image, 2 blocks contained one 1/3 morph image [derived from the block's identical face], and 2 blocks contained two 1/3 morph images), whereas in the other conditions, occasional repetitions were inserted (in each of the 1/3 morph, 2/3 morph, and different conditions, of the 6 blocks per condition, 2 contained no repetitions, 2 contained 1 repetition, and 2 blocks contained 2 repetitions). The experiment was run in 2 versions such that each subject did one of the versions for the upright experiment, and the other version for the inverted experiment. For each of the experiments (upright, inverted) the versions were counterbalanced across subjects. Each version consisted of 222 different male facial images made of 78 (6 sets × 13 faces per set) different original faces (of different men) and 144 (6 sets × 12 target faces per set × 2 morph levels [1/3 and 2/3]) morphed images. The difference between the versions was that in each version the source face for the morph of each set was a different face (out of the same 13 faces that composed the set). Therefore only the morphed images were different between the two versions. See Stimuli subsection for further details. Importantly, for a specific subject the morphed images were different in the upright and the inverted experiments since each subject did a different version for the upright and the inverted experiments.

The inverted morph experiment was identical to the upright morph experiment in all aspects except for the faces being inverted upside-down.

For each pair of images (K, J) in a block, for each color channel (R, G, B) separately, *Euclidean distance* was defined by $d(K,J)=\frac{1}{\sqrt{imsize}}\sqrt{\sum {\left(K-J\right)}^{2}}$ with K, J measured in (0,255) units. The block distance for each channel was defined as the average distance across all pairs (K, J) in the block. The average Euclidean distance was defined as the average distance over all blocks and all the 3 RGB channels.

### Visual localizer fMRI experiment

This experiment lasted 564 s and included 5 conditions: grayscale still photographs (500 × 500 pixels or 9° × 9° of visual angle) of upright faces, inverted faces, houses, common man-made objects, and geometric texture patterns. The inverted faces stimuli were created from the upright facial stimuli by a 180° rotation. The faces stimuli were different than those used in the morph experiments. Each condition was repeated 7 times in a controlled and counterbalanced block design paradigm. Each block lasted 9 s, with interleaving 6 s fixations between blocks. The first and last fixations lasted 12 and 15 s, respectively. A block consisted of 9 different stimuli; each stimulus was presented for 800 ms and was followed by a 200 ms fixation screen. A central white fixation point (0.07° × 0.07°) was present throughout the experiment. One or two repetitions of the same image occurred in each block. Subject's task was to report by a button press whether the presented stimulus was identical to the previous stimulus or not.

### Behavioral experiment

This experiment was aimed at defining the profile of difference perception according to the morph levels (0–45% in 5% steps, and 100%). Events lasted 3 s: 1200 ms of fast image presentation (117 ms picture + 83 ms fixation, 6 times) + 1800 ms fixation. Each event consisted of 6 stimuli. On trials with non-identical stimuli (5%–45% and 100% morph levels), all 6 presented images were different. For identical trials (0% morph) all 6 images were identical. On each trial subjects were asked to report “different” if any change at all was noticed between the 6 briefly presented face images or “same”—if no change was noticed. The event-presentation setup was similar to the one in the rapid event-related fMRI experiment in our previous report (Gilaie-Dotan and Malach, 2007). The experiment included 12 conditions (11 morph levels and fixation), each repeated 12 times in a counterbalanced order. The first and last fixations lasted 2 s each, altogether lasting 436 s.

### Data analysis: fMRI

fMRI data were analyzed with the BrainVoyager QX software package (R. Goebel, Brain Innovation, Maastricht, The Netherlands) and with complementary in-house software. The first 2 images of each functional scan were discarded. The functional images were superimposed on 2D anatomic images and incorporated into the 3D data sets through trilinear interpolation. The complete data set was transformed into Talairach space (Talairach and Tournoux, 1988). Preprocessing of functional scans included 3D motion correction, slice scan time correction, linear trend removal, and filtering out of low frequencies up to 3 cycles per experiment. No spatial smoothing was applied to the data. For display purposes only, the cortical surface of a normalized Talairach brain was reconstructed from a 3D SPGR scan. The procedure included segmentation of the white matter using a grow-region function, the smooth covering of a sphere around the segmented region, and the expansion of the reconstructed white matter into the gray matter. The surface of each hemisphere was then unfolded, cut along the calcarine sulcus and additional predefined anatomical landmarks on the medial side, and flattened (as can be seen in the flattened cortical maps in Fig. 5).

### Statistical analysis: fMRI

The statistical analysis was based on the general linear model (Friston et al., 1994). A standard hemodynamic response function (Two Gamma HRF function, 5 s to response peak, 15 s to undershoot peak) was applied to a boxcar predictor constructed for each experimental condition except fixation, and the model was independently fitted to the signal of each voxel. A coefficient was calculated for each predictor using a least squares algorithm.

### Regions of Interest: definition and analysis

Regions of interest (ROI) were defined for each subject separately using the visual localizer experiment data with minimum cluster size of at least 6 contiguous functional voxels with *p* < 0.05, uncorrected. Face-selective FFA and OFA regions were defined by upright faces > houses contrast. FFA was determined as the face-selective region in the posterior aspect of the fusiform gyrus (see Supplementary Material for alternative FFA definitions we have applied), and was defined for 11 of the 12 subjects. OFA was determined as the face-selective region residing in the lateral–occipital aspect of the cortex in the vicinity of the inferior occipital sulcus or gyrus (IOS or IOG respectively), and was defined in 11 of the 12 subjects. PPA and TOS place-selective regions were defined by houses > upright faces contrast. PPA was determined as the house-selective region residing in the parahippocampal gyrus or the adjacent collateral sulcus, while the dorsal transverse occipital sulcus (TOS) was determined as the house-selective region in the occipito-parietal aspect of the cortex beyond retinotopic cortex in the vicinity of the transverse occipital sulcus. PPA and TOS were each defined in all the 12 subjects. Object-selective LO was defined by the objects > textures contrast in the lateral–occipital aspect of the cortex in the vicinity of the inferior occipital sulcus or gyrus (IOS or IOG respectively) and was determined in all of the 12 subjects.

**Fig. 2:**
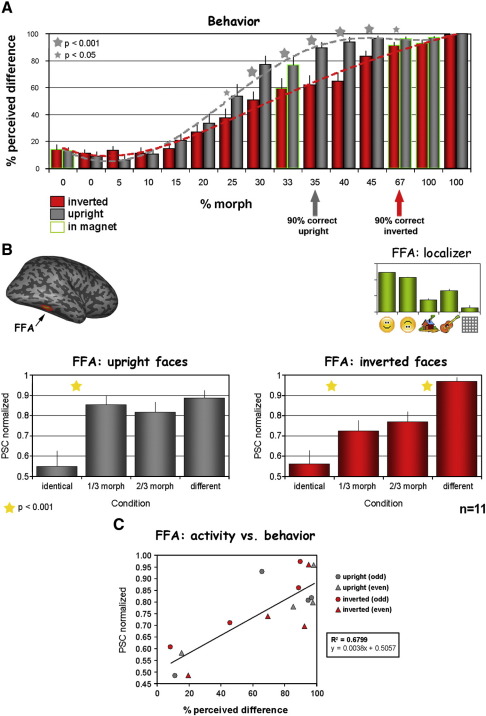
Behavior and FFA's adaptation effects. (A) Behavioral face discrimination for upright and inverted faces. The figure compares the measured ability of subjects to detect differences between the morphed faces (level of morphing on the *x*-axis) for upright (gray) and inverted (red) faces. The results displayed are combined from the psychophysical experiments and behavior during the scanning (delineated by green contour, see Results and Materials and methods for more details). Asterisks–for a specific morph level–indicate a significant difference between the discrimination performance of upright and inverted faces at this morph level (small = *p* < 0.05, large = *p* < 0.001, paired 2-tailed *t*-test on *n* = 12 subjects). Error bars denote S.E.M. Note the higher discriminability of upright faces compared to inverted ones, reflecting the well known “face inversion effect”. (B) FFA's adaptation profiles for upright and inverted faces (*n* = 11). Adaptation levels in the FFA at different morph levels are depicted for upright (left, gray) and inverted (right, red) faces. Note that the adaptation to inverted faces shows less shape selectivity (greater shape invariance) manifested in significant adaptation effects even at the 2/3 morph level. No such adaptation was found for the upright faces (left panel, gray). Note the similarity between the adaptation profiles and the behavioral discriminability (A) which supports the independent-neurons model (Fig. 1). Histogram on top right presents FFA's activation profile for the localizer experiment. Error bars denote S.E.M. Note the similar activation levels (as compared with the adaptation levels) to upright and inverted faces despite the significant differences in behavior. (C) Correlation between behavioral discrimination and fMRI adaptation in the FFA for upright and inverted faces. Each symbol specifies average perceived difference (*X* axis) vs. FFA's average normalized activation (*Y* axis). Gray (red) circles and triangles represent upright (inverted) faces data (over the odd or the even subjects respectively, *n* = 10). Regression line is denoted in black (its equation and the coefficient of determination (*R*^2^ = 0.6799 with *p* < 0.0001, non-directional) are displayed on the right). Note that the same analysis for the single subject data yielded a highly significant correlation as well with *R*^2^ = 0.3649, *p* < 0.001, non-directional (see Supplementary Fig. 4).

In order to obtain the activation and adaptation profiles of the MRI experiments for each region of interest (e.g. Fig. 2B for FFA, and see also Supplementary Material), the experimental time courses of activation were sampled from each subject's predefined ROI (see details above). These time courses are displayed in Supplementary Fig. 3. For each time course the percent signal change (PSC; relative to the preceding fixation block) over two time points along a block (for both morph experiments the time points were 9 s (3rd TR) and 12 s (4th TR) after stimulus onset, for the localizer experiment 6 s and 9 s after stimulus onset) was averaged, then this was averaged across all the repetitions of a specific condition. For each condition, the average response profile was calculated as the average across all subjects. Additional ROI adaptation profiles that are based on the peak response for each condition in each of the subjects are provided in Supplementary Fig. 2 accompanied by corresponding statistical analysis.

Since the extent of adaptation is a relative measure, in order to analyze the adaptation effects in a similar manner across subjects the PSC was normalized for each subject separately. This was done by dividing the PSC of each of the conditions in a specific experiment by the subject's maximal PSC over all the experiment's conditions. The normalized PSC presented in the Figures and text is the average of the normalized PSC over all the subjects. The statistical analysis that accompanies the normalized activation levels (ANOVA and post hoc tests) is based on individual subjects' normalized activation values. Supplementary Figs. 2 and 3 display the PSC (peak response histograms and full time courses) without the normalization procedure.

### Correlation analysis

The relationship between the perceptual similarity of the morphed faces and the adaptation tuning width was quantitatively estimated for each ROI by plotting the two parameters in a scatter plot (Fig. 2C, Fig. 4, Supplementary Fig. 4). For each condition the normalized PSC response (*y*-axis) was plotted against the perceived difference (*x*-axis). Single subject data are presented for the FFA in Supplementary Fig. 4. Fig. 2C data present average values of 2 sets of subjects (even or odd numbered subjects). Fig. 4 displays correlation and significance values for each region of interest.

### Multi subject analysis—Localizer fMRI experiment

In the multi-subject analysis, time courses of all subjects were converted into Talairach space and *z*-normalized. The multi-subject maps that are displayed in Fig. 5 were obtained using a random effect (RE) procedure (Friston et al., 1999). Statistical significance levels were calculated taking into account the individual voxel significance, a minimum cluster size of 6 functional voxels, and the probability threshold of a false detection of any given cluster within the entire cortical surface (Forman et al., 1995). This was achieved using a Monte Carlo simulation (AlphaSim by B. Douglas Ward, a software module in (Cox, 1996)). For visualization purposes the maps were projected on a flattened Talairach normalized brain.

### Statistical analysis: Behavior

In order to determine whether at a specific morph level there was a significant behavioral difference between upright and inverted faces, the subjects' discrimination results from both experiments were compared via a paired 2-tailed *t*-test. Significant results are displayed in Fig. 2A.

## Results

### Behavioral tuning

The fMR-adaptation experiment was run separately for upright and inverted faces, replicating the experimental paradigm we recently reported on (Gilaie-Dotan and Malach, 2007). Subjects' task was a one-back discrimination task in which they were required to report whether a face image was identical to or different from the previously presented face image via a response box (two alternative forced choice). Low-level motion cues and differences between subsequent images were controlled for (see Materials and methods for details). In addition, following the fMRI experimental scans, we measured the subjects' performance outside the scanner (behavioral experiment) with a similar experimental task to better quantify the perceptual difference detection levels along the morph axis for upright and for inverted faces.

In each of the two behavioral experiments (upright and inverted) subjects observed very brief sequences of varying degrees of morphing (ranging from 0% to 100% morph) and had to report whether they noticed a difference (“different”) or not (“same”). These behavioral results are presented in Fig. 2A. The behavioral curve for the upright faces (presented in gray) confirmed the narrow behavioral tuning curve we reported on earlier (Gilaie-Dotan and Malach, 2007), where at 30% morph there was a significant perception of face difference (difference perception of 77% ± 6%), and at 35% morph there was already a difference detection rate of 90% ± 4%. Importantly, the behavioral tuning curve for the inverted faces (indicated in red) was significantly broader compared to the upright faces starting from low-intermediate morph levels of 25% morph (*p* < 0.03, paired 2-tailed *t*-test). Note that this broader tuning showed a significant difference throughout all the intermediate morph levels and up to the 67% morph (*p* < 0.05 for 25%–67% levels, with *p* < 0.001 for 30%–45% levels, paired 2-tailed *t*-test), where the difference perception for the inverted faces reached a level of 90% correct. These differences were manifested in a steeper non linear increase in shape difference detection as a function of morph level during upright face presentation, as compared to a more gradual, monotonic increase for the inverted faces.

### Adaptation effects in the FFA

A primary aim of this study was to find the relationship between the FFA's adaptation effects to upright and inverted faces and the behavioral sensitivity for face differences. The location of the FFA was determined for each subject separately (upright faces > houses) using a separate localizer experiment. Fig. 2B depicts the adaptation profiles of the FFA to both upright (left; gray) and inverted faces (right; red). The activation profile of the FFA to the different categories in the localizer experiment can be seen on the top right of the panel. Upright faces showed a trend for somewhat higher activation compared to inverted faces (BOLD PSC, faces: 1.71 ± 0.1, inverted faces: 1.49 ± 0.1; see Supporting Material for further statistics on upright faces vs. inverted faces), while objects and houses activated this region to a much lesser extent. Activations to textures were close to baseline level (BOLD PSC, objects: 0.94 ± 0.14, houses: 0.54 ± 0.08, textures: 0.24 ± 0.13). As expected for the upright morphed faces experiment, the adaptation profile (left) was narrow and replicated our earlier results (Gilaie-Dotan and Malach, 2007), where in the 1/3 morph condition (33% morph level) there was a full release from adaptation. This was manifested in a significant signal increase in the 1/3 morph condition, while there was no significant difference between the activation levels of the 1/3 morph condition and the different-faces condition (identical vs. 1/3 morph: *p* < 0.001; 1/3 morph vs. different: *p* > 0.93 (Bonferroni/Dunn *post-hoc*); see further details in Supplementary Material).

A significant correspondence could be seen when comparing the behavioral shape tuning for upright faces with the corresponding fMR-adaptation profile (gray profile in Figs. 2A and B).

The critical issue however was whether FFA's adaptation profile for the inverted faces would follow the broader behavioral tuning profile (Fig. 2A red profile). As can be seen in Fig. 2B (right), the adaptation profile for the inverted faces was indeed broader than that of the upright faces. For the inverted faces we found a significant release from adaptation only in the 2/3 morph condition (Bonferroni/Dunn *post-hoc*: identical vs. 1/3 morph: *p* = 0.012 (N.S.); identical vs. 2/3 morph: *p* < 0.003; see Supplementary Material for further details), and unlike the upright faces, there was a significant difference between the level of activation to the 2/3 morph condition and the activation to the different-faces condition (2/3 morph vs. different: *p* < 0.005 (Bonferroni/Dunn *post-hoc*), see further details in Supplementary Material). Furthermore, when we directly compared the release from adaptation in the different morph conditions in the upright and inverted faces experiments (ANOVA with main factors: (i) face orientation, and (ii) morph level) we found that there was a significant interaction between face orientation and morph level (*p* < 0.05, *F* > 1), and *post-hoc* analysis revealed that for upright faces the release from adaptation was similar for the 1/3 morph and the different conditions (*p* > 0.93) whereas for inverted faces the release from adaptation in these two conditions was significantly different (*p* < 0.05).

To examine the possibility that the differences in the adaptation profiles between the upright and inverted faces were somehow related to the use of upright faces in the ROI definition (FFA, defined by upright faces vs. houses) we additionally examined alternative definitions of the FFA that might reveal higher selectivity to inverted faces. These results are presented in Supplementary Figs. 1A and 1B. It is clear that even when the FFA was determined by preference to inverted faces over houses (Supplementary Fig. 1A) or over textures (Supplementary Fig. 1B), the adaptation profiles were essentially unchanged (statistical analysis failed to reveal any significant differences between these profiles, *p* > 0.17, see Supplementary Material for more details).

Thus, in the fusiform gyrus the neuronal population displayed a different adaptation tuning width for the two categories being processed (upright or inverted faces). These tuning properties appeared to nicely and consistently correspond to the behavioral shape sensitivity in that narrower behavioral sensitivity for upright faces was reflected in a narrower adaptation recovery profile.

In order to quantitatively estimate the relationship between the perceived similarity of the morphed faces and the adaptation tuning width, we plotted the two parameters in a scatter plot. Fig. 2C depicts the results of this analysis (see Supplementary Figure 4 for same analysis based on single subject data). As can be seen in the Fig., the correlation between behavioral and fMR-adaptation selectivities revealed by this analysis was positive, strong and significant (Pearson correlation coefficient *R* = 0.8245, coefficient of determination *R*^2^ = 0.6799 with *p* < 0.0001, non-directional). This analysis reinforces the correspondence between the perceptual discrimination of the subjects and the fMRI adaptation effects in the FFA.

### Adaptation effects in additional regions

**Fig. 3:**
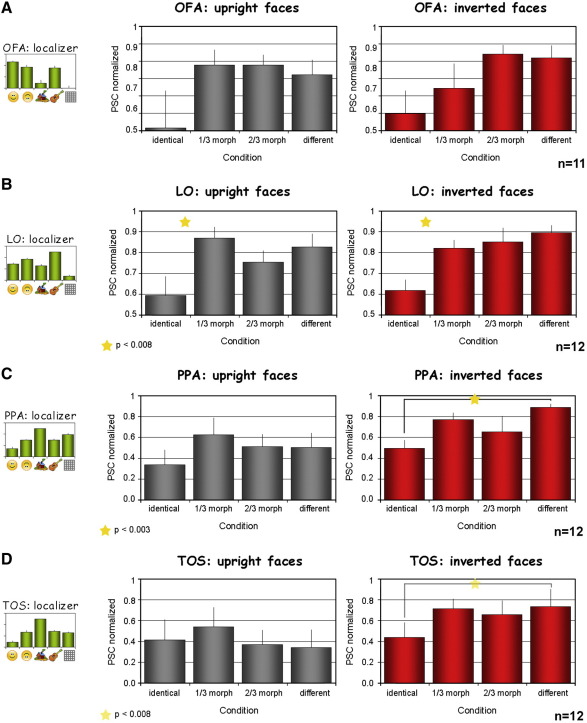
fMRI adaptation profiles of additional category selective regions. Histograms from the upright morphed faces, inverted morphed faces and localizer experiments (same presentation format as in Fig. 2B) for (A) face selective OFA, (B) object selective LO, (C) place selective PPA, (D) place selective TOS. See results for further details.

In addition to the FFA we examined the profiles of adaptation in additional predefined category-selective ROIs. As was done for the FFA, these regions were defined for each subject separately by the common functional definitions using the localizer experiment (see Materials and methods for further details). The borders of these regions, based on multi-subject analysis, are indicated by different colors in Fig. 5 for orientation purposes. The fMRI adaptation profiles of these regions, based on individual subject sampling, are shown in Fig. 3 (see Supplementary Fig. 2 and Supplementary Fig. 3 for adaptation profiles based on peak response and full time courses).

As can be seen in these figures, the face-selective occipital face area (OFA) displayed a pattern of adaptation that resembled that of the FFA (narrow tuning to upright faces while broader tuning to inverted faces, see Fig. 3A), albeit more noisy and not reaching significance (no main effects or interactions were found, upright faces: hemisphere: *F* < 1, *p* > 0.93, condition: *F* = 2.46, *p* = 0.096, interaction: *F* < 1, *p* > 0.72; inverted faces: hemisphere: *F* < 1, *p* > 0.73, condition: *F* = 2.961, *p* = 0.059, interaction: *F* < 1, *p* > 0.54).

Interestingly, in the object related lateral occipital (LO) area (defined by objects > textures) we found significantly narrow tuning to both upright and inverted faces, which for inverted faces was narrower than the discrimination performance (Fig. 3B). ANOVA analysis results for upright faces revealed significant effect for condition (*F* > 1, *p* < 0.01), and no other effects (hemisphere: *F* < 1, *p* > 0.81, interaction: *F* < 1, *p* > 0.47). *Post-hoc* analysis revealed narrow tuning to upright faces (Bonferroni/Dunn: upright identical-1/3morph: *p* < 0.002, 1/3morph not significantly different from 2/3morph (*p* > 0.17) or from different (*p* > 0.67)). For inverted faces we found a condition effect (*F* > 1, *p* < 0.003), no hemisphere effect (*F* < 1, *p* > 0.92), and no interaction (*F* < 1, *p* > 0.79). *Post-hoc* Bonferroni/Dunn: significant identical-1/3morph (*p* < 0.008), 1/3morph-2/3morph not significant (*p* > 0.74), and 1/3morph-different not significant (*p* > 0.29).

In the place-related regions, both ventrally in the parahippocampal place area (PPA, Fig. 3C) and dorsally in the transverse occipital sulcus (TOS) area (Fig. 3D) we found broad tuning to inverted faces, while for upright faces we found no significant difference between the identical and different face conditions. In the PPA for upright faces no effects were found (hemisphere: *F* < 1, *p* > 0.55, condition: *F* = 1.7, *p* > 0.18, interaction: *F* = 1.02, *p* > 0.39). Further analysis comparing strictly identical and different responses (1-tailed paired *t*-test) revealed a borderline difference (*p* = 0.078).

For inverted faces, only an effect of condition was found (*F* = 3.88, *p* < 0.02; hemisphere: *F* = 4.07, *p* = 0.068, interaction: *F* < 1, *p* > 0.56). *Post-hoc* for identical-1/3morph was only significant according to Fisher's PLSD but not by Bonferroni/Dunn: *p* = 0.0312, N.S., identical-2/3morph: *p* > 0.18, however identical-different was significant *p* < 0.003. When directly comparing the identical and different conditions we found a significant adaptation effect for the inverted faces (*p* < 0.0004, 1-tailed paired *t*-test).

Thus, it seems that the PPA was more sensitive to inverted faces than to upright faces. Observing the response amplitude difference in the localizer data also seems to indicate preferential activation to inverted compared to upright faces.

No effects were found for upright faces in the TOS (hemisphere: *p* > 0.63, condition: *p* > 0.57, interaction: *p* > 0.13) even when comparing directly identical and different faces condition (*p* > 0.31, 1-tailed paired *t*-test). For inverted faces a trend was found only for condition (*F* = 2.55, *p* = 0.072), hemisphere: *F* < 1, *p* > 0.85, interaction: *F* < 1, *p* > 0.81^2^. A direct comparison between identical and different conditions revealed a significant adaptation effect (*p* < 0.008, 1-tailed paired *t*-test).

**Fig. 4:**
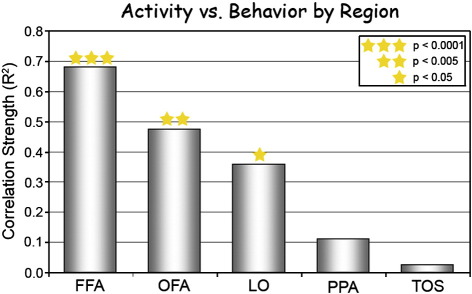
Correlation between behavioral discrimination and fMRI activation by region (based on the upright and inverted faces experiments' data together). Same analysis that was applied on FFA data in Fig. 2C, was applied here to additional ROIs. Correlation strength (*R*^2^) is represented on the *Y* axis. Asterisks above each bar indicate the significance of the correlation. These results are based on data from 10 subjects for FFA and OFA and on data from 11 subjects for LO, PPA and TOS. Note that the FFA shows the strongest correlation to behavior performance.

A correlation analysis as the one performed for the FFA (Fig. 2C), was performed for OFA, LO, PPA, and TOS as well. A summary of the correlation values (between activity and behavior) is presented in Fig. 4. Note that the FFA exhibited the strongest correlation to the behavior (Yovel and Kanwisher, 2005).

Thus, similar to what we found in the FFA, in these additional category-related regions we also observed that the same neuronal population had a different tuning width according to the category being processed.

### Differences in cortical activations to upright and inverted faces

**Fig. 5:**
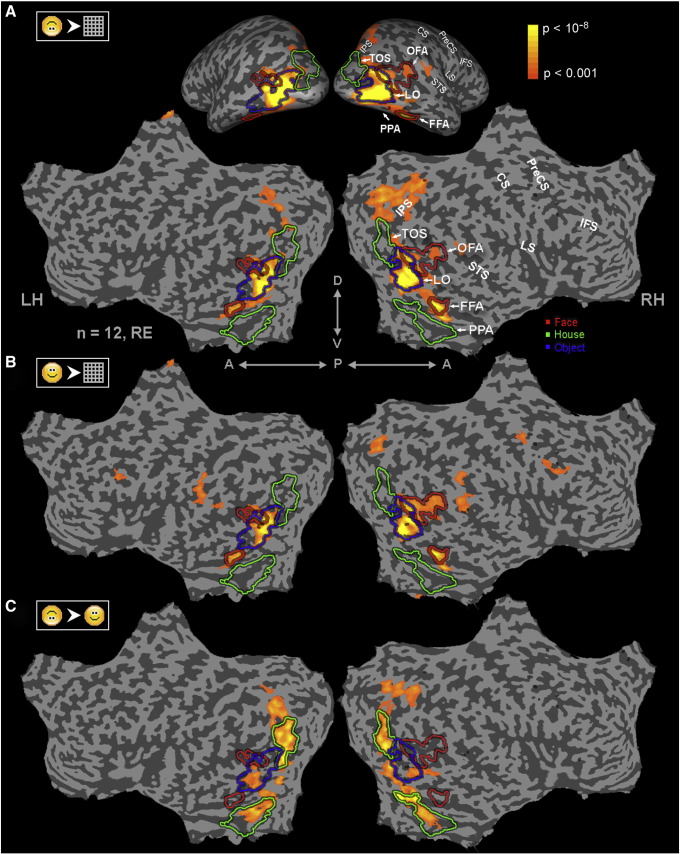
Upright and inverted faces maps. Multi-subject analysis (Random effects (RE), *N* = 12 subjects) of the localizer experiment is presented both in inflated hemisphere format (A (top), posterior view) and in unfolded cortical format (A (bottom), B, C). (A) High order areas activated by inverted faces. Yellow to orange patches denote regions that were activated above baseline and showed significantly higher activation to inverted faces over textures (*p* < 0.001 to *p* < 10^− 8^, corrected, see scale bar at top right). Colored lines represent the borders of category selective regions. Face-selective regions (red) were defined by faces vs. houses, place-selective regions (green) by houses vs. faces, and object-selective regions (blue) by objects vs. textures. (B) High order areas activated by upright faces. Yellow to orange patches denote regions that were activated above baseline and showed significantly higher activation to upright faces over textures (presentation format and statistical thresholds as in (A)). (C) Cortical areas showing preferential activation to inverted compared to upright faces. Yellow to orange patches denote regions that were activated above baseline and showed significantly higher activation to inverted faces over upright faces (presentation format and statistical thresholds as in (A)). Anatomical abbreviations: LH—left hemisphere, RH—right hemisphere, D—dorsal, V—ventral, P—posterior, A—anterior, FFA—fusiform face area, LO—lateral occipital, OFA—occipital face area, PPA—parahippocampal place area, TOS—transverse occipital sulcus, STS—superior temporal sulcus, IPS—intraparietal sulcus, LS—lateral sulcus (insula), CS—central sulcus, PreCS—precentral sulcus, IFS—inferior frontal sulcus. Note the extensive activation to inverted faces which includes most of the face-selective regions (red contour) and object-selective cortex (blue contour, cf. panel A and B) and extends dorsally. The preferential activation to inverted over upright faces was localized mainly to place and object-related cortex.

The localizer experiment that was conducted enabled us to also compare category-selective activations directly. This experiment included images of upright and inverted faces as well as man-made objects, houses and textures. The maps showing the main patterns of activations are presented in Fig. 5. The figure depicts the activation to the inverted and upright faces relative to the well known category-related regions in high order visual cortex (see panels A and B). More specifically, as can be seen in Fig. 5A, preferential activation to inverted faces relative to textures was evident in high-order visual cortex and included face-selective regions (FFA, OFA denoted by red borders), object-selective LO (denoted in blue) and parietal activations but did not extend into place-related regions (denoted in green). Similarly to inverted faces, upright faces (Fig. 5B) activated, in addition to face-related regions, object-related regions but not place-related regions. When observing the inverted faces' and upright faces' activation patterns compared to textures, it seems that they largely overlapped. Dissimilarities were observed in the more dorsal face-sensitive regions where upright faces activated OFA and STS to a greater extent, while inverted-faces activated parietal cortex to a greater extent.

However, when contrasting directly the activation patterns to inverted and upright faces (Fig. 5C), we find a surprisingly prominent preferential activation to inverted faces in mid-level visual areas overlapping place-sensitive representations as well.

## Discussion

### The perceptual inversion effect is reflected in fMRI adaptation

The main outcome of the present study is the observation that the behavioral face inversion effect (FIE), i.e. the reduced discrimination ability for inverted faces compared to upright ones was reflected in the tuning of adaptation effects in ventral stream high order visual areas, and particularly the fusiform gyrus (see Fig. 2). Specifically, we found that the fMRI adaptation effect was substantially more invariant to physical shape changes when these were imposed on inverted faces as opposed to upright faces (cf. Fig. 2B right and left panels). More generally, our results reveal that human shape discrimination ability as reflected in behavioral performance follows the tuning of the fMRI adaptation effects in high order visual areas.

### Can adaptation effects be informative about single unit activity?

The aim of this study was to use adaptation effects in order obtain an indirect measure of the tuning of the underlying neuronal population. However, it is still not fully established to what extent can neuronal tuning be accurately deduced from such an indirect measure. Recent single unit studies of the neuronal adaptation in primate infero-temporal cortex, have supported the notion that adaptation effects can be observed at the single cell level (Liu et al., 2009; Verhoef et al., 2008), and as expected, can be largely attributed to habituation or fatigue of neuronal inputs (McMahon and Olson, 2007; Sawamura et al., 2006). Similar manipulations of repetition duration in both macaque IT single cells study and fMRI human FFA study provide further evidence for the link between BOLD and neuronal adaptation (Fang et al., 2007; Liu et al., 2009). However under carefully selected conditions single unit adaptation effects have been shown to deviate from the fMR adaptation profile (McMahon and Olson, 2007; Sawamura et al., 2006). Thus, because of its indirect measure, and its dependence on averaged population activity, it is not possible, at present, to deduce a precise quantitative estimate of the neuronal tuning width from the adaptation effects (a complementary difficulty is of course inherent in single unit recordings which sample a small subset of the neuronal population). Consequently even under ideal conditions, one should not expect a precise correspondence between behavioral performance and the fMR adaptation profile. On the other hand, so far the adaptation method has proven the most sensitive measure of human neuronal selectivity—for example, in consistently showing exemplar sensitivity in ventral stream areas. Concerning the present study, even disregarding precise quantitative identity between adaptation profile and behavioral performance, our results show a significant correspondence between the two phenomena which is sufficient to provide important constraints on possible models of neuronal representations (see further below).

### Task-related influences

Could the adaptation effects reported here be related to the task—e.g. fatigue or reduced attention?

Our fMRI adaptation measures were obtained while subjects performed a perceptual discrimination task. It should be noted that shape discrimination of inverted faces was a far more difficult task and thus likely required more attentional resources, yet we found *less* signal recovery in the inverted case.

### Model predictions vs. the observed findings

The two extreme models presented in the introduction lead to contrasting experimental outcomes along two dimensions. One dimension is the relationship between perceptual discriminability and the sensitivity of single neurons. A straight forward prediction of the independent neuron coding model was that perceptual discriminability should correspond to the neuronal tuning selectivity. The second less obvious dimension, relates to magnitude of the neuronal representation engaged by each image—i.e. the number of responsive neurons. Note that in the independent neuron coding model, because the tuning width of neurons to the inverted faces is broader, it may paradoxically *increase* the number of activated neurons for the inverted face images and thus may compensate for lower activation of these neurons.

Considering the alternative extreme model, i.e. a population coding scheme, here the two predictions are inverted. While for the first dimension we expect no change in the tuning width of individual neurons associated with reduced perceptual discrimination, the lower perceptual performance for inverted faces could be reflected in the number of neuronal “channels”—i.e. it could be associated with a reduction in the *population size* of neurons activated by the inverted face images (e.g. akin to the reduction of color channels in color blindness).

Both aspects of the observed results, at least with regards to the FFA, are compatible with the independently tuned model:

First, considering the amplitude of the BOLD signal it is interesting to note that apart from a trend in the FFA (see Supplementary Material for further details), the fMRI response to inverted faces was of equal or even higher magnitude compared to upright faces (Fig. 5C). Similar results have been reported by a number of previous studies (Aguirre et al., 1999; Epstein et al., 2006; Haxby et al., 1999; Kanwisher et al., 1998), but see (Yovel and Kanwisher, 2005). These results illustrate that caution should be exercised when taking fMRI signal amplitude as a definitive marker of cortical specialization. As we have argued elsewhere (Avidan et al., 2002b; Gilaie-Dotan and Malach, 2007) the fMRI signal is a product of both the activation amplitude of individual neurons as well as the *number* of neurons that are activated. An increase in amplitude of activation of individual neurons could, for example, explain the reported correlation between fMRI amplitude in the FFA and the face inversion effect (Yovel and Kanwisher, 2005). However, the high fMRI signal in response to inverted faces may also be attributed to a larger number of neurons activated by each inverted face compared to an upright one.

Second, the tuning width of neurons, as reflected in the adaptation effect was significantly broader for the inverted compared to the upright faces. These results are compatible with previous studies (e.g. (Mazard et al., 2006; Yovel and Kanwisher, 2005)) showing higher adaptation for upright faces. In fact, while observing significant adaptation for upright faces, Yovel and Kanwisher did not find a significant adaptation for inverted faces. This accentuated difference in adaptation profile compared to the present study could be a result of differences in the experimental designs (e.g. block vs. event related design or different sets of face images).

In additional relevant research, a study using morphed famous faces (Rotshtein et al., 2004) has shown a tight correlation between the perceptual sensitivity to face identity changes and fMRI adaptation effects. A different study (Fox et al., 2009) has shown that adaptation effects in face-sensitive regions follow the perceived change in facial expression or identity. Finally, Welchman et al. (2005) have shown that perceived 3D shape changes from various cues are tightly linked to the BOLD adaptation effects in LOC and MT+. Together with the present results, all these studies are compatible with the notion that the adaptation profile in high-order visual cortex is coupled to the perceptual discrimination of the subjects.

It appears then that despite the caveats discussed above regarding the indirect nature of the adaptation method, the most parsimonious interpretation of the present results is in favor of the independently tuned model over the population model. Thus, our results suggest that the behavioral discriminability of faces can be attributed to the independent shape tuning width of *individual* neurons rather than some inter-neuronal computation performed at the population level by coarsely tuned neurons.

### Extension of our results to other object categories

Our results indicate that the independent-neurons coding principle is not unique to the highly familiar and specialized category of upright faces, but can be extended and generalized to unfamiliar visual categories such as inverted faces. Although inverted faces differ from common objects on many dimensions, the advantage of face inversion lies in the similar low-level composition of the upright and inverted faces, and consequently the ability to impose identical shape changes under conditions of drastically different perceptual discriminability. Furthermore, the similarity between the pattern of activation to inverted faces and the more general object-related activity (see Fig. 5) further supports the extension of the independent neurons coding scheme to more general object representations.

If indeed inverted faces can be considered as a general object category, then our results suggest that the difference between the representation of highly familiar and behaviorally significant objects such as upright faces and those of unfamiliar shapes lies in the *tuning width of individual neurons* rather than in the basic coding scheme of the neuronal representation. Further studies using additional object categories are needed to fully confirm this conjecture. While single unit recordings in human object representations have been so far unavailable to directly verify this conclusion, it is interesting to note that exquisitely narrow shape tuning has been well documented for single units involved in shape representation in monkey IT (Tanaka, 1996).

It is important to emphasize at this point that positing the highly complex neuronal representation of objects in terms of two extreme alternatives is an oversimplification. For example, the models assume that the representation is “holistic”—i.e. the basic representational elements are entire faces or at least large and complex object fragments. However, certainly at various stages of these representations, local image elements, such as informative “fragments”, also play an important role (Lerner et al., 2008). Similarly, recent evidence from single unit recordings in monkeys point to important contributions of certain face feature parameters such as iris size to the neuronal activation (Freiwald et al., 2009). However, while not ruling out these aspects, the present results highlight that an important component of the representation, particularly at high levels of the hierarchy such as the FFA, is a holistic representation in which neurons are tightly tuned to specific faces.

### A neuron of many faces

It may seem paradoxical how neurons can remain both sharply tuned in a holistic fashion to specific face templates yet at the same time belong to a distributed representation consisting of many, likely millions of neurons (Levy et al., 2004b). A possible solution to such conundrum is a unique type of a receptive field, which can be metaphorically thought of as a “totem pole” of different face templates. In other words, each neuron serves as an “OR” function and shows sensitivity to a large collection of different templates. However, the neurons are also sharply tuned to each template, so any change or deformation of the template's shape leads to drastic reduction in the neuronal activation. It is important to note that under such a scheme, where each neuron represents a multitude of *different* object images, the firing of each neuron in isolation is completely ambiguous, hence devoid of much informational value. However, making the plausible assumption that neurons differ in the exact constellation of images they are sensitive to, then the integrated activity of a neuronal assembly sharing a single common object template can fully resolve such ambiguity and constitutes a well defined representation of this specific object. It is easy to demonstrate that such a scheme allows a truly vast combinatorial space in which all possible object images both familiar and unfamiliar can be easily accommodated (Levy et al., 2004b) without the need of specific exemplar exposure or training.

To conclude, our results suggest a model in which the shape discrimination of human object representations can be traced to the responses of individual neurons. However, such fine discrimination at the level of single neurons can be accommodated only by allowing each neuron to represent, in a holistic manner, numerous different object templates.

### Compatibility with face representation models

The scheme we suggest for facial representations was focused on our ability to discriminate between different face or object images. However, it is important to clarify that the narrow tuning we report here for the shape of upright faces does not necessarily generalize to other image dimensions. For example, despite their narrow shape sensitivity, high order object representations display a relatively large invariance to changes in other image dimensions such as size, location, and contrast (Avidan et al., 2002a).

Another interesting dimension to consider is that of familiarity effects. Recent studies have introduced the concept of a “face space” (Bruce et al., 1991; Valentine and Endo, 1992) whereby an important dimension in recognizing the identity of faces concerns their morph distance from a “norm” or an average face (Leopold et al., 2001; Leopold et al., 2006; Rhodes and Jeffery, 2006). Note that in this formulation the movement from one face exemplar to another is orthogonal to the movement towards and away from the average face—and hence our study is not informative about this dimension. However, if human face representations indeed follow a face-space architecture, then our independent-neurons model leads to an interesting prediction: the adaptation profile should be much broader when morphing faces along the “identity” axis towards or away from the averaged face as compared to when morphing faces between different exemplars. A comprehensive, parametric study, in which faces will be morphed along various dimensions in face-space will be needed to fully clarify these significant issues.

## Footnotes

2 Although not significant, we did run the *post-hoc* analysis to test which differences contributed to the condition effect. The identical-1/3morph did not pass Bonferonni/Dunn post-hoc significance (*p* = 0.0265, N.S.), but was found significant by Fisher’s PLSD post-hoc test (*p* = 0.0265), where 1/3morph was not significantly different from complete release (1/3morph-2/3morph: *p* > 0.57, 2/3morph-different: *p* > 0.49).
