# Supplementary material for: Perceptual shape sensitivity to upright and inverted faces is reflected in neuronal adaptation
Source: Neuroimage. 2010 Apr 1;50(2-3):383–95. doi: 10.1016/j.neuroimage.2009.12.077 (PMC3221039; doi:10.1016/j.neuroimage.2009.12.077)
Supplement: Supplementary material [file mmc1.pdf]

Perceptual shape sensitivity to upright and inverted faces is reflected in neuronal adaptation

Sharon Gilaie-Dotan, Hagar Gelbard-Sagiv, Rafael Malach

Supplementary Material

Statistical analysis of the FFA's adaptation effects

The data analysis was performed on the FFA time courses as defined for each subject separately based on the category localizer experiment.
The following analysis is based on time courses that were sampled from regions in the posterior fusiform gyrus that showed preference to *upright faces over houses,* and this definition is the one that is referred to in the Results.

Narrow tuning of the adaptation effects to upright faces

In general, regardless of the sampling strategy for defining the FFA (as revealed here in this analysis and in additional control FFA definitions we applied as described further on), the pattern that emerges in the FFA for upright faces was consistent and indicates essentially *complete recovery of the signal in the 1/3morph condition* to a similar level as that of the different condition.

A repeated measures ANOVA with condition (identical, 1/3morph, 2/3morph, and different) and hemisphere (right and left) on the time courses of 11 subjects revealed a significant **effect for condition ($F > 1$, $p < 0.002$)**, no effect for hemisphere ($F < 1$, $p > 0.57$), and no interaction ($p > 0.18$). A further post-hoc analysis for condition revealed a significant **identical-1/3morph** difference (Bonferroni/Dunn: **$p < 0.001$**), and no significant effects for 1/3morph-2/3morph ($p > 0.57$) or 1/3morph-different ($p > 0.93$).

Broad tuning of the adaptation effects to inverted faces

In general, across the different definitions of the FFA the pattern that emerges for inverted faces was that there were 2 *significant steps in the recovery of the signal* from adaptation. Critically, in all cases the signal was not completely recovered in the 1/3morph condition, indicating a broader tuning than that found for upright faces.

A repeated measures ANOVA with condition (identical, 1/3morph, 2/3morph, and different) and hemisphere (right and left) on the time courses of 11 subjects revealed a significant effect for **condition ($F > 1$, $p < 0.0001$)**, no significant effect for hemisphere ($p > 0.088$), and no interaction ($p > 0.45$). A further post-hoc analysis for condition revealed a close to significant identical-1/3morph difference (Bonferroni/Dunn: $p = 0.012$ (N.S. under this test)). Significant release from adaptation was reached at the 2/3morph (**identical-2/3morph**: $p < 0.003$). Critically, **2/3morph-different** difference was significant (Bonferroni/Dunn: $p < 0.005$), indicating essentially incomplete recovery of the signal at 2/3morph.

Alternative control definitions of the FFA and adaptation analysis

In order to expose neuronal populations that might be sensitive to inverted faces and that were "shaded" by the classical FFA definition favoring upright faces representations (faces vs. houses), we repeated the fMRI adaptation analysis for upright and inverted faces as sampled from two alternative definitions of the FFA that might expose those higher selectivities to inverted faces.

First alternative definition was based on preference to *inverted faces over houses*. The FFA was identified in 12 subjects using this definition (in 10: bilaterally, in 1: right only, in 1: left only). Data from this analysis are presented in Supp. Fig. 1A. Repeated measures ANOVA with condition (identical, 1/3morph, 2/3morph, and different) and hemisphere (right and left) on the time courses of 10 subjects (with bilateral foci) essentially replicated the results derived from the classically defined FFA:

In the *upright faces* experiment: A significant effect for **condition ($F > 1$, $p < 0.0002$),** no effect for hemisphere ($F < 1$, $p > 0.83$), and no interaction ($F = 1.583$, $p > 0.21$). A further post-hoc analysis for condition revealed a **significant identical-1/3morph difference** (Bonferroni/Dunn: $p < 0.0004$), and no significant effects for 1/3morph-2/3morph ($p > 0.96$) or 1/3morph-different ($p > 0.45$).

In the *inverted faces* experiment: A significant effect for **condition ($F > 1$, $p < 0.0001$)**, no significant effect for hemisphere ($p > 0.2$), and no interaction ($F = 1.01$, $p > 0.4$). A further post-hoc analysis for condition revealed a **significant identical-1/3morph** difference (Bonferroni/Dunn: $p < 0.004$). **1/3morph - different was significant (Bonferroni/Dunn: $p < 0.002$),** indicating incomplete recovery of the signal at 1/3morph.

Second alternative definition was based on preference to *inverted faces over textures* (Supp. Fig. 1B). Here also the alternative definition of the FFA did not alter the previously described adaptation profiles. Repeated measures ANOVA with condition (identical, 1/3morph, 2/3morph, and different) and hemisphere (right and left) on the experiment time courses of 11 subjects (with bilateral foci) revealed:

In the *upright faces* experiment*:* A significant effect for **condition ($F > 1$, $p < 0.0001$)**, no effect for hemisphere ($F < 1$, $p > 0.28$), and no interaction ($F = 1.83$, $p > 0.16$). A further post-hoc analysis for condition revealed a significant **identical-1/3morph** difference (Bonferroni/Dunn: $p < 0.0001$), and no significant effects for 1/3morph-2/3morph ($p > 0.77$) or 1/3morph-different ($p > 0.25$).

In the *inverted faces* experiment: A significant effect for **condition ($F > 1$, $p < 0.0001$)**, no significant effect for hemisphere ($F = 2.74$, $p > 0.12$), and no interaction ($F < 1$, $p > 0.49$). A further post-hoc analysis for condition revealed a significant **identical-1/3morph** difference (Bonferroni/Dunn: **$p < 0.0008$**). 1/3morph condition was not significantly different from the 2/3morph (Bonferroni/Dunn: $p > 0.26$), but was significantly different from the different condition (Bonferroni/Dunn: $p < 0.0002$) as was the **2/3morph-different** difference (Bonferroni/Dunn: $p < 0.003$).

Furthermore, we applied statistical analysis to the different FFA profiles in order to determine whether a statistical difference between these profiles could be detected. The single-subject BOLD activations levels sampled from the three different FFA definitions (from both the upright and inverted experiments) were submitted to a 3-way repeated measures ANOVA ($3_{\text{FFA definitions}} \times 2_{\text{face-orientation experiments (upright, inverted)}} \times 4_{\text{experimental conditions}}$). No significant effects or interactions were found between the 3 activation profiles of the FFA with respect to FFA's definition ($p > 0.17$, $F = 1.93$; interactions: definition by orientation: $p > 0.42$, definition by condition: $p > 0.27$, 3-way interaction: $p > 0.72$) indicating on a very consistent FFA tuning profile across the 3 FFA definitions.

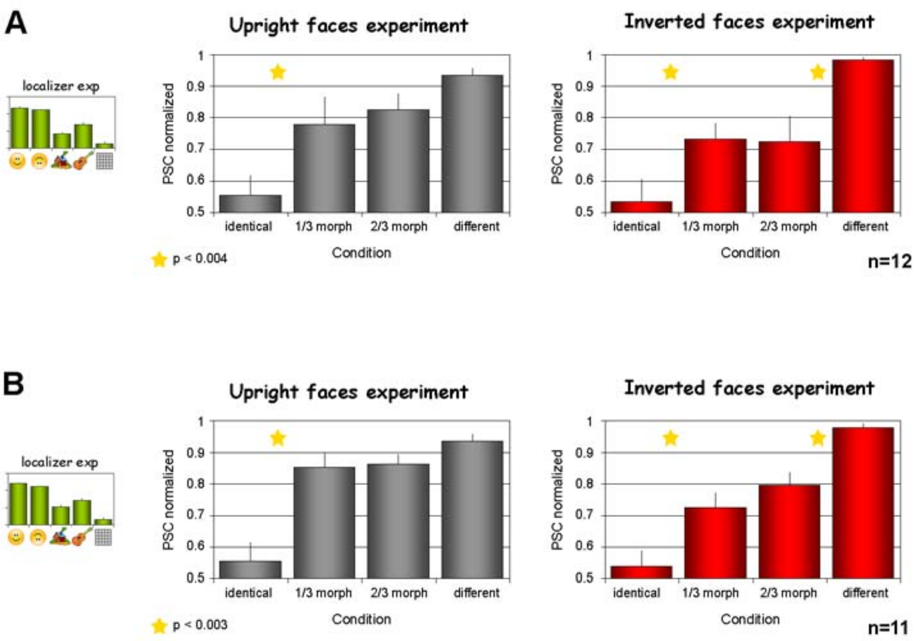

Supp. Fig. 1. Alternative definitions of the FFA do not affect adaptation profiles. fMRI adaptation profiles of the FFA when defined by *inverted faces over houses* **(A)** or by *inverted faces over textures* (B). Left: localizer results, middle: upright faces results, right: inverted faces results. Error bars, S.E.M. Note that these alternative definitions for the FFA resulted in the same adaptation profiles as we got from the classical definition of the FFA (cf. Fig. 2B).

FFA's response magnitude to upright and inverted faces in the category localizer experiment

When FFA was defined by the faces vs. houses contrast, a 1-tailed paired t-test across subjects showed that upright faces activated this region more than inverted faces ($p < 0.002$). However, note that this test was biased towards upright faces and not independent of the FFA's definition. When FFA was defined by inverted faces vs. houses, no significant activation difference was found between upright and inverted faces ($p > 0.34$).

Adaptation profiles derived from peak responses

In our original analysis for each block we used the average PSC over two pre-defined time points (3rd and 4th TRs in morph experiments, 2nd and 3rd TRs in localizer) as the representative PSC for that block. Here we repeated the analysis using a single time point with the highest PSC (peak response) as the representative for the block response. Results presented in Supp. Fig. 2 replicated our original results. Detailed below are results from a repeated measures 2-way ANOVA with condition (identical, 1/3morph, 2/3morph, and different) and hemisphere (right and left) based on peak responses of each subject to each of the conditions:

FFA (n=11)

Upright faces experiment: A significant effect for **condition ($F = 6.09$, $p = 0.0023$)**, no effect for hemisphere ($F < 1$, $p > 0.75$), and no interaction ($F = 1.65$, $p > 0.19$). A further post-hoc analysis for condition revealed a significant **identical-1/3morph** difference (Bonferroni/Dunn: $p = 0.0017$), and no significant effects for 1/3morph-2/3morph ($p > 0.67$) or 1/3morph-different ($p > 0.71$).

Inverted faces experiment: A significant effect for **condition ($F = 13.646$, $p < 0.0001$)**, no significant effect for hemisphere ($F = 3.24$, $p = 0.102$), and no interaction ($F = 1.05$, $p = 0.385$). A further post-hoc analysis for condition revealed significant differences for **identical-2/3morph**[1] (Bonferroni/Dunn: **$p = 0.0067$**) and **2/3morph-different** (Bonferroni/Dunn: $p = 0.0019$).

OFA (n=11)

Upright faces experiment: No effects or interactions were significant (hemisphere: $F < 1$, $p > 0.88$, condition: $F = 1.87$, $p = 0.17$, hemisphere X condition: $F = 0.696$, $p > 0.56$). When right and left data were combined a 1-way ANOVA revealed a significant effect for **condition ($F = 3.088$, $p = 0.042$).** A further post-hoc analysis for condition revealed a significant **identical-1/3morph** difference (Bonferroni/Dunn: $p = 0.0083$), and no significant effects for 1/3morph-2/3morph ($p = 0.409$) or 1/3morph-different ($p = 0.633$).

Inverted faces experiment: A significant effect for **condition ($F = 5.023$, $p = 0.0088$)**, no significant effect for hemisphere ($F = 0.922$, $p = 0.369$), and no interaction (F =

[1] Identical – 1/3 morph was not significant according to Bonferroni/Dunn ($p = 0.0341$)

0.785, p = 0.516). A further post-hoc analysis for condition revealed no significant identical-1/3morph effect (Bonferroni/Dunn: p = 0.052), a significant **identical-2/3morph** difference (Bonferroni/Dunn: **p < 0.0008**). 2/3morph condition was not significantly different from the different condition (Bonferroni/Dunn: p > 0.41).

LO (n=12)

Upright faces experiment: A significant effect for **condition (F = 4.07, p = 0.015)**, significant effect for **hemisphere (F = 4.85, p = 0.0499)**, and no interaction (F = 2.03, p = 0.129). A further post-hoc analysis for condition revealed a significant **identical-1/3morph** difference (Bonferroni/Dunn: p = 0.0032), and no significant effects for 1/3morph-2/3morph (p > 0.24) or 1/3morph-different (p > 0.74).

Inverted faces experiment: A significant effect for **condition (F = 4.92, p = 0.0062)**, no significant effect for hemisphere (F = 2.9, p > 0.11), and no interaction (F = 2.52, p = 0.075). Further post-hoc analysis for condition revealed a significant **identical-2/3morph** difference (Bonferroni/Dunn: **p = 0.006**), identical-1/3morph was not significant (Bonferroni/Dunn: 0.0447). 2/3morph-different was not significant (Bonferroni/Dunn: p >0.5).

PPA (n=12)

Upright faces experiment: No significant effects (condition: F = 1.17, p = 0.336, hemisphere: F = 2.776, p = 0.124) or interaction (F = 1.72, p = 0.183).

Inverted faces experiment: A significant effect for **condition (F = 3.793, p = 0.0193)**, significant effect for **hemisphere (F = 6.066, p = 0.0315)**, and no interaction (F = 1.39, p = 0.264). A further post-hoc analysis for condition revealed a significant **identical-different** difference (Bonferroni/Dunn: **p < 0.0023**); not significant were identical-1/3morph (p = 0.0322), identical-2/3morph (p=0.0849).

TOS (n=12)

Upright faces experiment: No significant effects (condition: F = 1.55, p = 0.221, hemisphere: F = 0.081, p = 0.782), but significant interaction (F = 3.74, p = 0.0203) which was further qualified as between conditions difference in the left hemisphere only (paired 2-tailed t-test: 1/3morph vs. different, p = 0.034, and 1/3morph vs. 2/3morph at p = 0.085, identical-different at p = 0.075). In the right hemisphere all comparisons yielded p > 0.38.

Inverted faces experiment: No significant effects (condition: $F = 1.218$, $p = 0.319$, hemisphere: $F = 0.01$, $p = 0.923$), or interaction ($F = 1.33$, $p = 0.283$) were found.

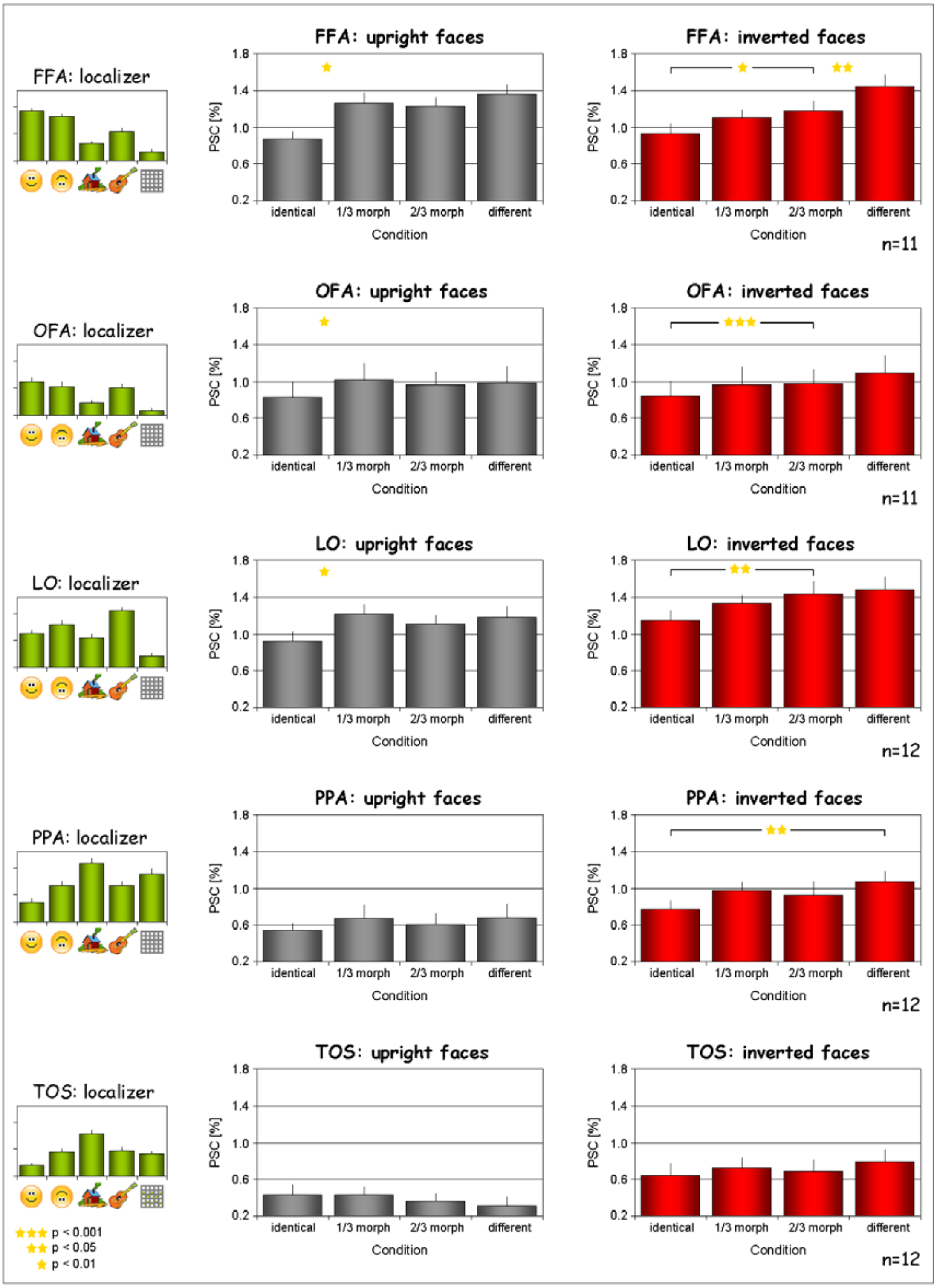

Supp. Fig. 2. fMRI adaptation profiles of category selective ROIs, *based on peak responses* (instead of average response over several time points). Histograms for localizer (left), upright morphed faces (middle) and inverted morphed faces experiments (right) are presented. Conventions as in Fig, 3.

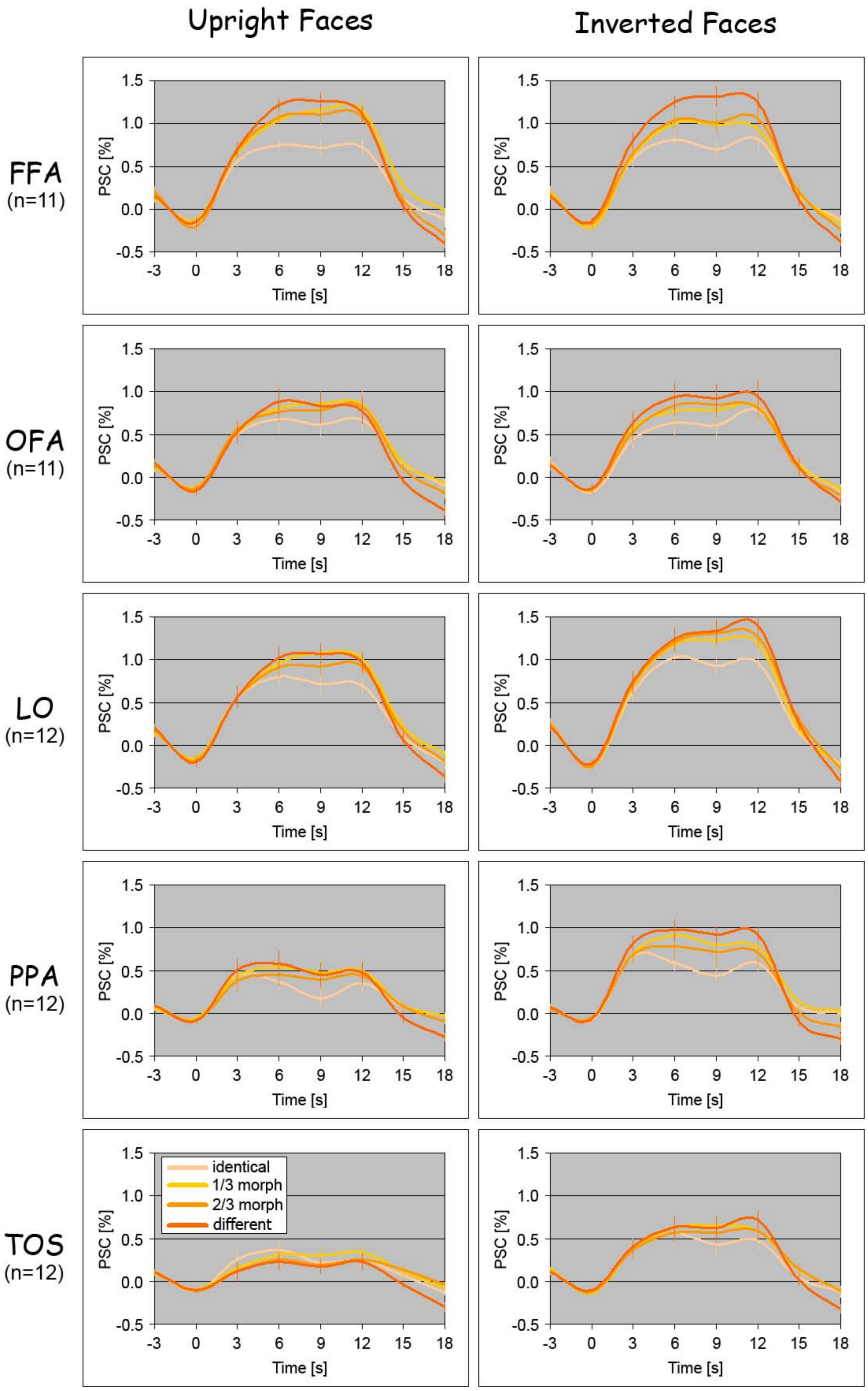

Supp. Fig. 3. Average time courses of category-related ROIs (from top to bottom: FFA, OFA, LO, PPA, TOS). For each region the upright faces time courses are displayed on the left, inverted faces time courses on the right. Identical condition is

presented in light pink, 1/3morph in yellow, 2/3morph in light orange and different condition in orange (see figure legend imposed on TOS upright histogram). For each condition in each of the ROIs, the average response profile was calculated as the average across all the subjects relative to the fixation period baseline. Values are displayed in percent signal change units. Error bars denote S.E.M. See further details in methods. The number of subjects denoted for each region is the number of subjects this region was identified in successfully.

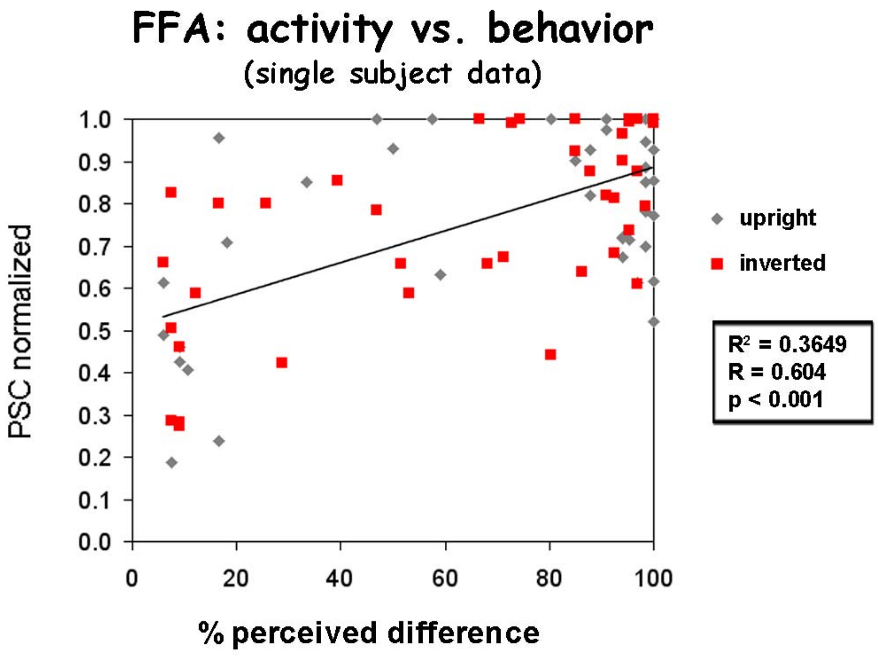

Supp. Fig. 4. Correlation between behavioral discrimination and fMRI adaptation in the FFA for upright and inverted faces based on single subject data (n=10). Each symbol specifies average perceived difference (X axis) vs. FFA's average normalized activation (Y axis). Gray dots represent upright faces data (4 dots per subject over the four conditions, n=10). Red dots represent the inverted faces data. Regression line denoted in black (its coefficient of determination (R^2 = 0.3649, $p < 0.001$, non-directional, equation $y = 0.0037x + 0.5116$) is displayed on the right).
